# Meeting of the German Naturalists and Physicians at Berlin

**Published:** 1886-12

**Authors:** 


### MEETING OF THE GERMAN NATURALISTS AND PHYSICIANS AT BERLIN.

---

Reported for the Independent Practitioner.

---

The fifty-ninth meeting of the Society of the German Naturalists and Physicians was held in Berlin, from the 16th to the 25th of September, 1886. For the first time since the founding of the society, a dental section was organized. This, of course, was a natural sequel of the establishment of the dental institute as a department of the University of Berlin, and it is only to be wondered at that there could be a question raised as to the recognition of the dental profession in America, where so many universities have dental departments in connection with them.

The very condensed report which follows deals only with the questions discussed in the dental section, the sessions of which were attended by about seventy members.

Professor Busch, in charge of the clinic for extraction, called for an expression of opinion as to the advisability of extracting the two cuspids, when all other teeth are wanting, for the purpose of securing a better fitting plate.

Nearly all present agreed that perfectly normal cuspidati should never be extracted. When they departed from normality, either in length, color, position or firmness, the indications for extraction appear and increase in proportion to the abnormality.

Herr Herbst spoke about the rotation method. He was very much elated over his reception in America, and said that the rotation method stood as firmly as anything could stand, and was looked upon as the method of the future. It did not receive the hearty applause which was bestowed upon it in America, and the subject was discussed very little, the general impression being that nothing is to be gained by debating the question, for time only will show whether there is anything in the rotary method of introducing gold to warrant its general introduction.

Herr Herbst filled a large cavity involving the mesial and grinding surfaces of the right upper second bicuspid. Considerable time was required for preparing and adjusting the ring matrix, an operation which was accompanied by considerable pain. The filling was completed in about an hour. It appeared to be a good filling, but not perfect in every respect. It was evident to those who had seen Herbst operate two or three years ago, that the rotation method had undergone some changes. One who watched the operation throughout computed that during three-fourths of the time hand-pressure was used, not only for finding weak spots, but for condensing the gold. The use of heavy foils is also a new feature in the rotation method as now practiced by Herbst. There seems to be a great reluctance on the part of his German colleagues to take up the rotation method, and some who have tried it have confessed their inability to master it, and have returned to the popular methods.

Herbst then described a method of lining cavities with gold before filling with amalgam. (See INDEPENDENT PRACTITIONER, August, '86.)

Dr. Witzel stated that the method was employed by Siegmondi twenty years ago, and described in the journals of that date.

Dr. Hillischer confirmed the statement of Witzel. The method had been discarded because experience had shown that teeth so filled became discolored, the amalgam contracting and drawing the gold away from the walls. He much preferred to line weak cavities with cement. It gave support to the walls, and was not drawn away from them by the contracting of the amalgam.

Prof. Miller, of Berlin, spoke about the combination of tin and gold as a filling material. He claimed for it the advantages already stated in the INDEPENDENT PRACTITIONER, Vol. V, page 403. He showed a large filling on the grinding surface of a molar made in two and one-half minutes, a compound grinding and approximal filling made in seven minutes, and a tooth-crown, two walls only standing, built up with tin and gold and capped with gold in twenty-five minutes.

Dr. Richter, of Berlin, showed a tooth filled by Miller with tin and gold five years ago, which was recently extracted to correct an irregularity. The pulp was very nearly exposed at the time of filling, so that the tooth was not thoroughly excavated. The dentine had, however, become very hard, and the filling, which was now a hard mass, adhered firmly to the walls of the tooth. Dr. Sachs, Prof. Sauer, Prof. Hollaender, Dr. Göttinger and others, who had used the material, expressed their complete accord with Miller's views as to the ease of insertion and permanency of the tin and gold filling.

Herr Julius Parreidt, of Leipzig, read an article on cysts of the teeth and jaws. He combated the view of Magitot, that all cysts of the jaws are necessarily tooth-cysts. They can be formed in the jaw as well as in other bones, though this seldom happens. Parreidt retains the classification of Magitot in follicular and periosteal cysts; he had treated twenty-five cases, of which twenty-three were periosteal and two follicular. Periosteal cysts arise from a chronic irritation of the pericementum. The treatment consists in the removal of the tooth and keeping the cavity clean, the cyst healing of itself. The follicular cysts require an operation.

Prof. Busch remarked that he had seen twenty periosteal cysts at the clinic, but had not yet met with a case of follicular cyst.

Dr. Hillischer, of Vienna, spoke about narcosis from a mixture of oxygen and nitrous oxide. He had applied the mixture in nearly 1,000 cases, and had secured better results than could be obtained with nitrous oxide alone. He thought it less dangerous, and hoped, by being able to continue the inhalation for a long time, that the mixture would eventually be employed for extended surgical operations. He exhibited an apparatus for inhaling the mixture under normal pressure, as well as for mixing the two gases at the time of inhaling, in any desired proportion. The mixture must not be allowed to stand any length of time, since the higher oxides of nitrogen are formed.

Prof. Busch was much pleased with the apparatus, but doubted if the mixture could ever be employed for long operations. Oxygen always diminishes the narcotic power of nitrous oxide.

Prof. Miller spoke on the restoration of the contour of carious teeth by means of porcelain. He mentioned three cases in which porcelain could be employed to advantage as filling material:

1. On the labial surface of incisors and cuspids.
2. For restoring the corner or entire cutting edge of incisors and cuspids.
3. For restoring the external walls of bicuspids.

In the first case, the piece of porcelain is first fixed in oxy-phosphate cement, and then filled around with gold; in the second and third cases gold is not necessary. Prof. Miller made a number of operations of the second and third class in 1879, which are still holding perfectly well. He recommended the method very highly, especially for cases of the second class. The method of preparing the cavities and grinding the porcelain pieces and inserting them, were explained and demonstrated.

Prof. Sauer exhibited a new apparatus for correcting protruding upper jaws. The object of the contrivance was to raise the articulation so that the lower incisors do not occlude with the upper, the lip then pressing the teeth back without further aid.

Dr. Eysell, of Cassel, spoke on the contraction of the nasal cavity produced by the narrowness of the palate and abnormal position of the teeth.

Herr Ritter, of Berlin, read an article on Antiseptics in Dentistry. He called attention to the fact that numerous cases of suppurative inflammation, necrosis, etc., might be avoided by a proper attention to the antiseptic treatment of wounds produced in the mouth by extraction or otherwise. He is in the habit of disinfecting the gums before each case of extraction, and afterwards, particularly in unclean mouths, of treating the wound antiseptically.

Prof. Miller called attention to different pathogenic micro-organisms which he had found in the mouth, and agreed with Ritter, that wounds in the oral cavity should be treated with the same precaution as to antiseptic conditions which are taken in treating wounds in any other part of the body.

Drs. Witzel, Schreiber and Hillischer were of the same opinion.

Prof. Sachs thought that the treatment required too much time, the object being to relieve the patient of pain and anxiety as quickly as possible.

Prof. Busch spoke of the preparation of tooth-sections. He had tried the various methods in use, and had experienced great difficulty in cutting teeth, particularly those with folds of enamel. He now uses a thin metallic disk, which, while in rotation, is fed with a paste of corundum powder and water. With this the hardest teeth may easily be cut.

Prof. Busch further spoke on supernumerary and missing teeth of the human denture. He expressed the opinion that most supernumerary teeth are to be regarded as originating in fragments of tooth-germs. Individual cases might, in accordance with the theory of descent, be looked upon as a tendency to return to the original type.

Herr Morgenstern, of Baden-Baden, presented a short communication on tooth-grafting, and introduced a patient for whom he had, seven years previously, performed the operation with great success.

Prof. Miller passed around a photo-micrograph, showing the manner in which the attachment of the reimplanted tooth takes place. He laid great stress upon the necessity of preserving the pericementum, since the true attachment can take place through that alone.

Herr Zimmermann related the case of a tooth which was extracted by mistake, and in the hurry to get it back again was put in "wrong side to." The operation was, however, an entire success, owing to the perfect preservation of the pericementum.

Prof. Sauer was in favor of resorting to reimplantation only in extreme cases.

Dr. Cunningham, of Cambridge, was of the same opinion. He described a splint for reimplanting teeth, consisting of a mass of oxy-phosphate cement pressed in a soft state around the reimplanted and the neighboring tooth, and held with the thumb and finger till it hardens. He referred to experiments which showed that a reunion takes place between the vessels of the pulp and those of the subjacent tissue.

Herr Morgenstern stated that this was not the case. The pulp mummifies, and a pseudo-pulp grows into the root-canal through the apical foramen.*

Herr Warnekras, of Berlin, gave his experience in the use of cocaine for extracting teeth. He injects half of a Pravasz syringe full of 20% solution, in three portions, the first along the inner root (or inner side of a one-rooted tooth), the second along the outer root, the third and largest portion into the vascular tissue at the point of the root. He then waits five minutes before extracting. He claims to have accomplished wonders in this way. He made three extractions at the clinic, which were not painless but in which the pain was evidently reduced.

It was urged against the use of cocaine that the extraction is not completely painless, and that the injection itself is quite painful, and, most of all, that it is dangerous. Many cases was cited where intoxication with alarming symptoms was produced by the injection of much less than one decigram (the amount used by W.).

*This is undoubtedly the case with dogs' teeth, which have a very large foramen. Further experiments may be necesssary to prove that it is also true of human teeth.—*Rep.*

